# Missense and Non-Missense Lamin A/C Gene Mutations Are Similarly Associated with Major Arrhythmic Cardiac Events: A 20-Year Single-Centre Experience

**DOI:** 10.3390/biomedicines12061293

**Published:** 2024-06-11

**Authors:** Cinzia Forleo, Maria Cristina Carella, Paolo Basile, Eugenio Carulli, Michele Luca Dadamo, Francesca Amati, Francesco Loizzi, Sandro Sorrentino, Ilaria Dentamaro, Marco Maria Dicorato, Stefano Ricci, Rosanna Bagnulo, Matteo Iacoviello, Vincenzo Ezio Santobuono, Carlo Caiati, Martino Pepe, Jean-Francois Desaphy, Marco Matteo Ciccone, Nicoletta Resta, Andrea Igoren Guaricci

**Affiliations:** 1 Cardiology Unit, Interdisciplinary Department of Medicine (DIM), University of Bari “Aldo Moro”, University Hospital Consortium Polyclinic of Bari, Piazza G. Cesare 11, 70124 Bari, Italy; m.carella31@studenti.uniba.it (M.C.C.); pabas2304@gmail.com (P.B.); e.carulli93@gmail.com (E.C.); dadamomicheleluca@yahoo.it (M.L.D.); francesca.amati86@gmail.com (F.A.); loizzi91@gmail.com (F.L.); sandrosorrentino@hotmail.com (S.S.); ilaria.dentamaro@gmail.com (I.D.); mm.dicorato@gmail.com (M.M.D.); s.ricci17@studenti.uniba.it (S.R.); vincenzoezio.santobuono@uniba.it (V.E.S.); carlo.caiati@uniba.it (C.C.); drmartinopepe@gmail.com (M.P.); marcomatteo.ciccone@uniba.it (M.M.C.); andrea.guaricci@gmail.com (A.I.G.); 2 Internal Medicine Section, Department of Precision and Regenerative Medicine and Ionian Area (DiMePRe-J), University of Bari “Aldo Moro”, University Hospital Consortium Polyclinic of Bari, Piazza G. Cesare 11, 70124 Bari, Italy; 3 Medical Genetics Unit, Department of Precision and Regenerative Medicine and Ionian Area (DiMePRe-J), University of Bari “Aldo Moro”, University Hospital Consortium Polyclinic of Bari, Piazza G. Cesare 11, 70124 Bari, Italy; rosanna.bagnulo@uniba.it (R.B.); m.iacoviello3@gmail.com (M.I.); nicoletta.resta@uniba.it (N.R.); 4 Pharmacology Unit, Department of Precision and Regenerative Medicine and Ionian Area, School of Medicine, University of Bari Aldo Moro, 70124 Bari, Italy; jeanfrancois.desaphy@uniba.it

**Keywords:** LMNA-related cardiomyopathy, non-missense mutation, arrhythmic risk stratification, implantable cardioverter defibrillator, gene–phenotype correlation

## Abstract

Arrhythmic risk stratification in patients with Lamin A/C gene (LMNA)-related cardiomyopathy influences clinical decisions. An implantable cardioverter defibrillator (ICD) should be considered in patients with an estimated 5-year risk of malignant ventricular arrhythmia (MVA) of ≥10%. The risk prediction score for MVA includes non-missense LMNA mutations, despite their role as an established risk factor for sudden cardiac death (SCD) has been questioned in several studies. The purpose of this study is to investigate cardiac features and find gene–phenotype correlations that would contribute to the evidence on the prognostic implications of non-missense vs. missense mutations in a cohort of LMNA mutant patients. An observational, prospective study was conducted in which 54 patients positive for a Lamin A/C mutation were enrolled, and 20 probands (37%) were included. The median age at first clinical manifestation was 41 (IQR 19) years. The median follow-up was 8 years (IQR 8). The type of *LMNA* gene mutation was distributed as follows: missense in 26 patients (48%), non-frameshift insertions in 16 (30%), frameshift deletions in 5 (9%), and nonsense in 7 (13%). Among the missense mutation carriers, two (8%) died and four (15%) were admitted onto the heart transplant list or underwent transplantation, with a major adverse cardiovascular event (MACE) rate of 35%. No statistically significant differences in MACE prevalence were identified according to the missense and non-missense mutation groups (*p* value = 0.847). Our data shift the spotlight on this considerable topic and could suggest that some missense mutations may deserve attention regarding SCD risk stratification in real-world clinical settings.

## 1. Introduction

Lamins are nuclear type V intermediate filament proteins underlying the inner nuclear membrane and are classified as A or B type. A-type Lamins (Lamin A and C) are encoded by the Lamin A/C gene (LMNA), which is located on chromosome 1 (1q21.2–q21.3) [1]. Lamins A and C have several pleiotropic effects that may explain why their dysfunction can lead to multiorgan involvement. First, they exert a structural function ensuring nuclear shape, size, and envelope stiffness; moreover, they actively participate in the organization of chromatin and modulation of nucleus–cytoskeleton connections, thereby regulating gene expression [2,3].

Patients with LMNA mutations express a variety of pathological phenotypes [4,5,6,7]. Most of them result in cardiomyopathies, while the others affect striated muscle, adipose tissue, and the peripheral nervous system, involving multiple tissues or leading to progeroid/overlapping syndromes [8].

LMNA-related cardiomyopathy (LMNA-CMP) has a complex phenotype, characterized by both mechanical and electrical abnormalities. In detail, it manifests with progressive dilated cardiomyopathy (DCM), atrioventricular conduction disturbances, and atrial and/or ventricular tachyarrhythmias, generally leading to terminal heart failure, sudden cardiac death (SCD), or stroke [9,10].

Pathogenic variants are mainly missense and nonsense mutations, while a few sporadic small deletions/insertions have been identified [11]. Haploinsufficiency has been proposed as a disease mechanism in patients carrying truncating variants [11]. In the literature, truncating mutations have been associated with an earlier onset of cardiac conduction defects, atrial arrhythmias, and lower left ventricular ejection fraction, compared with missense mutations [11]. However, there are currently no clear data demonstrating a strict relationship between the LMNA mutation type and a specific phenotype. Disease severity and prognosis show significant interindividual variability, not only among unrelated probands, but also within members of the same family [4].

The evidence on arrhythmic risk stratification in patients with LMNA-related DCM and hypokinetic non-dilated cardiomyopathy (HNDCM) is constantly evolving [12], as evidenced by the proposal of a new disease category, non-dilated left ventricular cardiomyopathy (NDLVC), introduced by the recent ESC Guidelines on cardiomyopathies [13]. This novel phenotype identifies morphological entities with the presence of non-ischaemic left ventricular (LV) scarring or adipose replacement, irrespective of the presence of wall motion abnormalities or isolated global LV hypokinesia without scarring [13]. NDLVC encompasses a phenotypic spectrum with cardiac borderline features that do not fit within the standard definitions of other well-defined cardiomyopathies [13,14,15,16].

**Figure 1:**
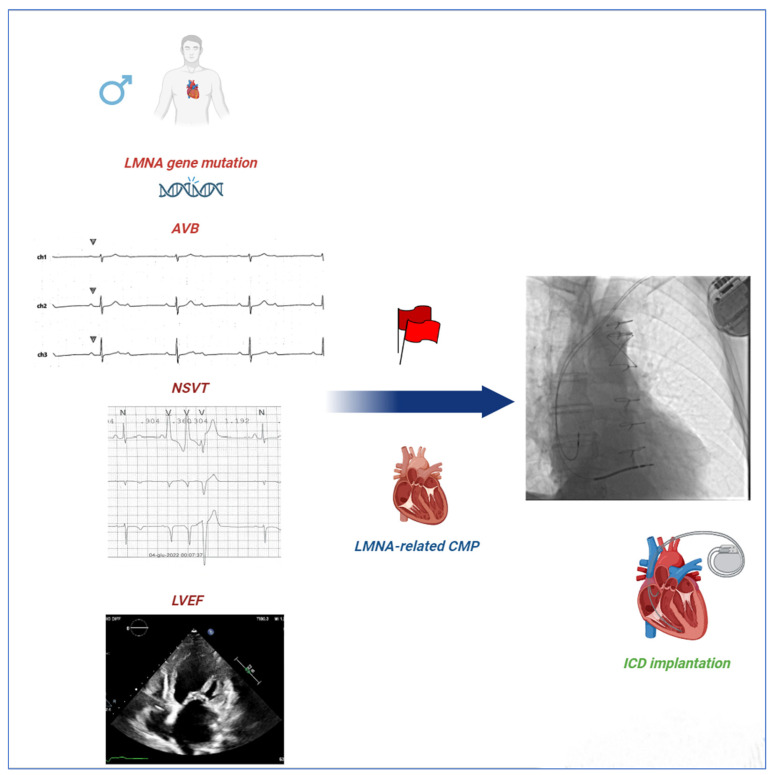
**Red Flags in LMNA-related CMP.** Wahbi’s score is the most used algorithm for predicting the risk of malignant ventricular arrhythmia (MVA) in patients with LMNA-related CMP. The algorithm includes several red flags such as male sex, non-missense LMNA mutations, NSVT, LVEF < 45%, and first- or greater-degree AVBs. ICD implantation is recommended for a value of 10% or higher. Our study raises concerns about the implications of non-missense mutations for prognosis compared to missense mutations in subjects with LMNA-linked CMP. AVBs = atrioventricular blocks; CMP = cardiomyopathy; ICD = implantable cardioverter defibrillator; LMNA = lamin A/C gene; LVEF = left ventricular ejection fraction; and NSVT = non-sustained ventricular tachycardia.

Knowledge on this critical topic can guide clinical decisions concerning implantable cardioverter defibrillator (ICD) therapy, which is the only intervention that can prevent SCD occurrence in patients affected by cardiomyopathies [17,18,19,20,21]. In this regard, the risk prediction score for malignant VA (MVA) in Laminopathies is based on the Wahbi model, which includes male gender, non-missense LMNA mutations (insertion, deletion, truncations, or mutations affecting splicing), NSVT, LVEF < 45%, and first- or higher-degree atrioventricular block (AVB) (Figure 1) [22]. However, the notion that only non-missense mutations represent an established risk factor for SCD in LMNA mutation carriers has been questioned in several studies [23,24,25,26].

Starting from these premises, we sought to investigate cardiac features and find gene–phenotype correlations that would contribute to the evidence on the prognostic implications of non-missense vs. missense mutations in a cohort of LMNA mutant patients.

## 2. Materials and Methods

### 2.1. Study Design

The study enrolled probands and family members carrying pathogenic or potentially pathogenic variants in the *LMNA* gene, who were referred to and periodically followed from January 2002 to January 2022 in our Cardiomyopathy Unit at a tertiary university hospital. We collected clinical and instrumental data retrospectively, starting from the first medical contact of each patient. The participants were then followed up prospectively according to a specific schedule. Clinical onset was defined as the time of the first clinical manifestation involving the heart or the skeletal muscles in relation to the LMNA disease.

The project was approved by the Ethics Committee and was conducted according to the Declaration of Helsinki and its later amendments. All patients or their guardians provided their written informed consent to participate in this study.

### 2.2. Baseline Assessment

At the time of enrolment in the study, all patients underwent a clinical cardiological evaluation consisting of personal and family anamnestic data collection, the monitoring of vital signs and analysis of symptoms (syncope, palpitations, chest pain, dyspnoea, and NYHA functional class), and laboratory tests including creatine kinase levels, 12-lead electrocardiogram (ECG), transthoracic echocardiogram, and 24 h ECG Holter monitoring, as previously described [26]. Where appropriate, coronary angiography was performed. Subjects without contraindications, such as severe claustrophobia or pacemaker/ICD not compatible with magnetic resonance imaging, underwent cardiac magnetic resonance.

Furthermore, for cardiac implantable electronic device (CIED) (ICD or cardiac resynchronization therapy with a defibrillator) (CRT-D) carriers, the device itself was checked and programmed. The patients with a CIED underwent remote monitoring.

Pharmacological therapy, including beta-blockers, angiotensin-converting enzyme inhibitors (ACE-i)/Angiotensin II type 1 receptor blockers (ARBs)/Angiotensin receptor-neprilysin inhibitors (ARNI), mineralocorticoid receptor antagonists (MRAs), sodium-glucose co-transporter 2 inhibitors (SGLT2i), diuretics, and antiarrhythmics, was given per standard of care, guided by the patient’s clinical status and in accordance with the international guidelines for the management of chronic and acute heart failure [27,28]. ICDs were implanted following the indications of the international guidelines for ventricular arrhythmia treatment and the prevention of SCD [14,15,16].

The baseline neuromuscular evaluation consisted of a detailed and standardized neurological examination with dedicated diagnostic tests, as previously indicated [26].

### 2.3. Follow-Up

After the first evaluation, all patients underwent standardized clinical follow-up visits at our clinic, at variable time intervals depending on the needs of the case, but at least once semi-annually, until the end of the study. Each monitoring examination included: clinical assessment, 12-lead ECG, transthoracic Doppler echocardiogram, 24 h ECG Holter monitoring, and ICD interrogation, where applicable. If clinically required, further assessments were performed.

### 2.4. Endpoints

The primary combined endpoint consisted of major adverse cardiac events (MACE), defined as a combination of cardiac death, heart transplantation, sustained VT, ventricular fibrillation (VF), VT/VF cardiac arrest, SCD, or appropriate treatment (anti-tachycardia pacing or shock) from an ICD. The secondary endpoints were: AVB of any grade, supraventricular (including atrial fibrillation/flutter) or ventricular tachyarrhythmias (encompassing premature ventricular complexes and NSVT), or any structural or functional echocardiographic abnormality [29].

### 2.5. Genetics

The sequencing of the *LMNA* gene by the Sanger technique was performed according to standard protocols. Molecular analyses were carried out at the Medical Genetics Unit, Department of Precision and Regenerative Medicine and Ionian Area, University of Bari Aldo Moro, University Hospital Consortium, Polyclinic of Bari, Italy. The identified variants were classified in accordance with the recommendations of the American College of Medical Genetics and Genomics and the Association for Molecular Pathology [30]. Based on their type, the *LMNA* mutations were divided into missense and non-missense mutations. The latter group included non-frameshift insertions, frameshift deletions, and nonsense mutations.

### 2.6. Statistical Analysis

All statistical analyses were performed using the Statistical Package for Social Sciences version 25 (SPSS, Inc., Chicago, IL, USA). Graphical analysis was performed using Rstudio IDE 2022.12.0 built 353. For continuous variables, normal distribution was evaluated using the Shapiro–Wilk test, and the values are expressed as mean and standard deviation or median and interquartile range (IQR) when appropriate. Differences in mean values were compared by t-tests for normally distributed data. In the case of non-normally distributed variables, a non-parametric Mann–Whitney test was used. Categorical variables were expressed as percentage values and compared by Pearson’s chi-squared test or Fisher’s exact test, where appropriate. The risk of cardiovascular adverse events between carriers of missense and non-missense mutations was evaluated by odds ratios. A *p* value of <0.05 was set for statistical significance.

The data, analytic methods, and study materials will be made available to other researchers for the purposes of reproducing the results or replicating the procedure.

## 3. Results

### 3.1. Study Population

The study enrolled 54 patients with LMNA mutations from 20 families (range, from 1 to 8 participants per family) and included 20 probands (37%) and 34 family members (63%). Twenty-nine of them were males (54%). Among the probands, 15 patients were male (75%). The median age at first clinical manifestation was 41 (IQR 19) years, while it was 45 [IQR 17] years at enrolment. A family history of cardiomyopathy was present in 45 participants (83%), sudden cardiac death in 29 subjects (54%), and arrhythmias in 30 (55%). The baseline characteristics are shown in Table 1.

At the time of the enrolment, all probands had electrical and/or mechanical abnormalities related to the *LMNA* gene variant.

The type of *LMNA* gene mutation was distributed as follows: missense in 26 patients (48%), non-frameshift insertions in 16 (30%), frameshift deletions in 5 (9%), and nonsense in 7 (13%) (Table 1).

### 3.2. Patient Cardiac and Neuromuscular Features

The first documented manifestation of the disease (in both probands and relatives) was cardiological in 41 participants (76%) and neuromuscular in 3 cases (5%), while the remaining patients were phenotype negative at enrolment. During the follow-up, it emerged that the median age at the onset of cardiological symptoms associated with cardiac electrical/mechanical alterations was 38 years (IQR 22), while it was 43 years (IQR 33) for musculoskeletal manifestations, without a statistically significant difference (*p* value = 0.849) (Table 1).

Musculoskeletal involvement was identified in nine (17%) of our patients, and in three (5%) of whom, it was recognized as the first manifestation of the pathology, before any cardiological sign (Table 1). Among patients with neuromuscular manifestations, the specific type 1B limb–girdle muscular dystrophy (LGMD1B) phenotype was diagnosed in two of them during the follow-up (Table 1).

Regarding MACE, no statistically significant difference between genders (43% in males versus 21% in females, *p* value = 0.081) was documented (Table 2). Tachyarrhythmic events occurred in both sexes with a similar frequency. In terms of AVB, males had a higher risk of high-grade blocks (OR 6.37, *p* = 0.016). Another notable finding of our study was that male subjects were at a greater risk of developing a dilated cardiac phenotype (OR 6, *p* = 0.003). No sex-related differences were demonstrated for left ventricular dysfunction (Table 2).

The median follow-up from study enrolment to the last clinical evaluation was 8 years (IQR 8). All living participants received their last follow-up assessment no more than 6 months before the end of the study.

### 3.3. Outcomes

At the end of the follow-up period, 44 participants (81%) showed signs of cardiac involvement, 9 cases (16%) developed skeletal-muscle manifestations, while 4 patients (7%) were phenotypically asymptomatic (Table 1).

Three patients (5%) died at the ages of 33, 60, and 76 years during the follow-up due to thromboembolic and arrhythmic complications; all of them experienced antecedent arrhythmic events. A total of six patients (11%) underwent heart transplantation, while two (4%) were added to the transplantation list.

Among the 44 patients affected by the cardiovascular abnormalities, it was possible to differentiate an arrhythmic and a structural phenotype.

In regard to the electrical and arrhythmic features, it emerged that AVB of any degree occurred in 26 patients (48%), but only in 13 (24%) was it of a high degree. Supraventricular arrhythmias (atrial fibrillation, flutter, or tachycardia) were identified in 22 cases (41%), premature ventricular complexes (PVCs) in 41 (76%), NSVT in 36 (67%), sustained VT in 16 (29%), ventricular fibrillation in 1 (2%), and appropriate ICD intervention in 17 (31%) of them (Table 1).

Among the structural cardiac phenotypes, LV dilation was observed in about one half of patients examined (28 patients, 52%), and 22% (12 patients) of them developed severe LV dysfunction. On the other hand, right ventricle dilation and/or disfunction involved 13 patients (24%).

Of the 44 patients with cardiovascular involvement, the combined phenotype (with both arrhythmic and structural anomalies) occurred in 32 participants (59%).

During the entire enrolment period, 46 (85%) reported being symptomatic, in most cases due to palpitations or dyspnoea, while only a few of them (4 cases) reported syncope.

**Figure 2:**
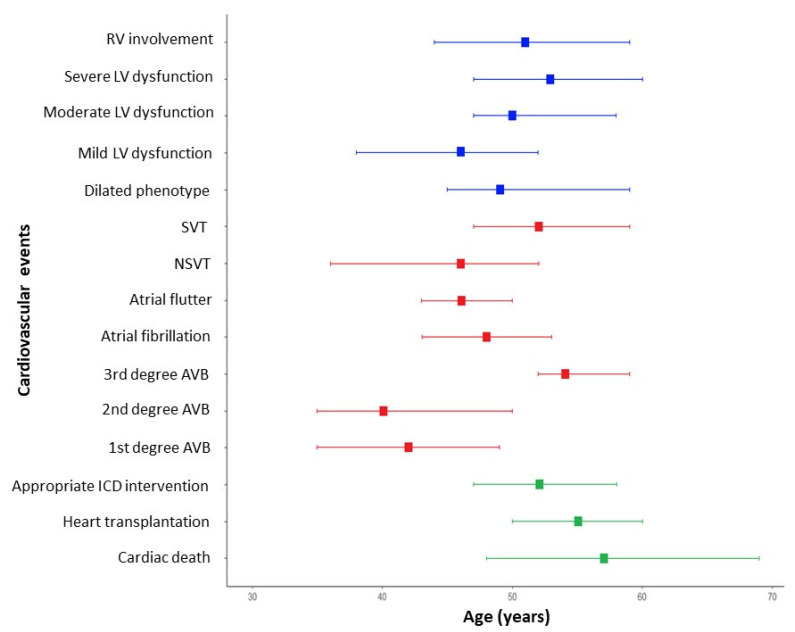
**Natural history and event timeline for patients with LMNA-related disease.** The timeline presents major and minor events in patients with LMNA-related disease, categorized by age of onset. Structural cardiac changes are represented in blue, in red colour brady and tachyarrhythmic events, while major cardiovascular events are displayed using green colour. AVB = atrioventricular block; ICD = implantable cardioverter defibrillator; LMNA = lamin A/C gene; LV = left ventricular; NSVT = non-sustained ventricular tachycardia; RV = right ventricular; and SVT = sustained ventricular tachycardia.

Each cardiac manifestation was assessed the median age of onset (Table 3 and Figure 2). It emerged that bradyarrhythmias such as AVBs of a lower degree were the earliest cardiologic manifestations (median age of the first degree AVB was 41 years—IQR 35–49), together with NSVT and mild LV dysfunction. A more relevant LV dysfunction, the dilated phenotype, and worrisome arrhythmic events arise later in the clinical course of the disease.

### 3.4. Missense vs. Non-Missense Mutations Comparison

A deeper analysis of the rate of cardiovascular manifestations according to missense vs. non-missense mutation types did not highlight any relevant differences between groups (Table 4). In the 26 missense mutation carriers (48% of cases), the rate of MACE was 35% compared to 32% in the non-missense group (*p* value = 0.847). Cardiac death was observed in two missense mutation patients (8%), and only one case in the non-missense population, without a statistical difference (*p* value = 0.509). Moreover, no statistically relevant differences between the missense and non-missense groups were observed in regard to ventricular and atrial tachyarrhythmias, (86% vs. 65% and 71% vs. 58%, respectively). There was no statistically significant difference (61% vs. 43%, *p*-value = 0.170) in the development of a dilated phenotype between the two groups.

Patients with non-missense mutations did not appear to have a different risk of bradyarrhythmias such as AVB compared to those with missense mutations (61% vs. 35%, *p*-value = 0.055). In addition, our analysis showed that first-degree AVBs were more frequent in non-missense variant carriers (61% vs. 31% respectively, *p* value = 0.027). In contrast, for second- or third-degree AVBs, there was no statistically significant difference (*p* value = 0.150) (Table 4).

## 4. Discussion

LMNA-related CMP is characterized by significant inter-individual variability in terms of disease severity and prognosis, not only between unrelated probands, but also within members of the same family. Currently, there is no clear evidence demonstrating a direct correlation between LMNA variants and specific phenotypes.

Our study focused on the cardiac features of patients with Lamin A/C mutants, considering the relationship of mutation type (missense vs. non-missense) with phenotypic profile, including electrical and arrhythmic characteristics, heart structural abnormalities, and MACE.

A strength of our study is that the data were collected in real-world clinical practice during an adequate duration of follow-up (median 8 years). In this regard, our survey reflects a real-life setting.

The first noticeable result was that the median ages at first clinical manifestation and at the time of enrolment were not significantly different (*p* value = 0.797). This underlines that the patients consulted our centre, in most cases, were already symptomatic or even with manifested cardiovascular involvement. The above data might be translated into clinical practice, suggesting the need for a greater effort by the treating clinicians in trying to provide probands and their families with the correct information on the importance of early screening for this disease. In the diagnostic process and therapeutic management of patients with LMNA mutations, the first and fundamental step is to raise laminopathy suspicion. This can guide further testing and lead to an early diagnosis, which is crucial in preventing the development of clinically evident disease [31,32]. Early diagnosis also allows for timely therapeutic measures to be taken, which can help to delay disease progression [27,28,33] and prevent SCD [14,15,16]. This process is often challenging due to the presence of clinical manifestations shared with other cardiac diseases [31].

The main result emerging from our study was the non-statistically significant difference between non-missense and missense mutations in regard to MACE (32% versus 35%, respectively, *p* = 0.847). This finding differs from some of the data available in the literature, which previously reported worse composite cardiac endpoints in both male and non-missense-mutation-harbouring patients [34,35,36].

A median follow-up of 57 months in a retrospective study of 94 LMNA mutation carriers revealed that non-missense mutations were associated with higher rates of ICD interventions and SCD compared to missense mutations, with 46% of non-missense mutation carriers experiencing events (*p* = 0.027). Splice site mutations were significant predictors of SCD (HR: 2.05; *p* < 0.001) [35].

In a European cohort of 269 LMNA mutation carriers, non-missense mutations significantly predicted MVA, defined as SCD, cardiopulmonary resuscitation, or appropriate ICD intervention, with a hazard ratio of 2.5 (95% CI: 1.4 to 4.5) [36].

A 7-year multicentre study of 122 patients also found that non-missense mutations were independently associated with the new onset of sustained ventricular arrhythmias (HR: 2.5; *p* = 0.03) [34].

Our results were consistent with those of other recent reports [23,24,25,26].

In a median follow-up of 10 years, an observational study was conducted on 164 LMNA mutation carriers from 13 centres of the Italian Network for Laminopathies [26]. The study did not find any association between male gender or non-missense mutations and a composite cardiac endpoint, which included cardiac death, heart transplant, and malignant ventricular arrhythmias [26]. This contradicts previous reports.

Similarly, the REDLAMINA registry, which followed 140 Spanish carriers for 5 years, identified NSVT and LVEF < 45% as the only independent predictors of major arrhythmic events, with no significant impact from gender or mutation type [23]. Interestingly, the REDLAMINA registry authors highlighted that some LMNA missense variants could be considered as low-risk variants, being associated with a late presentation and a good prognosis, while other LMNA missense variants could be assessed as high-risk mutants, since they are related to an unfavourable prognosis.

Furthermore, a study including 41 LMNA mutated patients who were followed for 29 months found no association between the type of mutation (missense vs. non-missense) and ventricular arrhythmias, defined as NSVT, SVT, and VF (*p* value = 0.93) [24].

Lastly, in a multicentre cohort of 77 LMNA mutation carriers followed for a median 4 years, the rate of all-cause cardiac mortality was comparable between the non-missense mutation group and the missense mutation one (12% vs. 11%, respectively). Moreover, although the cardiac phenotype in non-missense mutation carriers develops earlier, no significant difference in the penetrance of the disease was found (100% in the non-missense group vs. 90% in the missense group) [25].

Previous reports have proposed that the position of the mutation within the *LMNA* sequence might be related to the clinical phenotype [25,37,38]. Hegele et al. found a significant association between mutations located upstream of the nuclear localization signal (NLS, residues 416 to 423) and laminopathies with cardiac involvement (HR: 8.4; *p* < 0.0001) [37]. This association remained significant when considering only missense mutations. The region upstream of the NLS includes the Lamin rod domain, crucial for nucleoskeleton integrity, while the downstream region involves gene expression regulation [39].

Captur and colleagues analysed published LMNA mutations linked to Lamin heart disease, finding that mutations upstream of the NLS are associated with more severe cardiac phenotypes compared to downstream mutations (*p* = 0.014, OR 2.38) [40,41]. Interestingly, they suggested that not all missense variants share the same outcome, and some missense mutations could be as dangerous as non-missense mutations [40,41].

Recently, in a prospective cohort of 185,990 unrelated middle-aged UK Biobank participants, it was highlighted that both the predicted functional impact and location of rare genetic variants in *LMNA* are important for predicting cardiac disease susceptibility [38]. Over a median follow-up of 10.9 years, subjects carrying rare missense *LMNA* variations developed arrhythmias and cardiomyopathies at higher rates (HR: 1.95 [95% CI: 1.41–2.71]; *p* < 0.001) in comparison with non-carriers. Remarkably, missense variants located upstream of the *LMNA* NLS were strongly linked with cardiac phenotypes, allowing the authors to propose this Lamin region as a possible therapeutic target [38].

Regarding mutation site, in the present study, 52 mutations (96%) were located upstream, and 2 (4%) mutants were localized downstream of the NLS. These data could suggest that other parameters beyond the type of mutation are important, but prospective multicentre verification is needed.

Another finding that stands out from our analysis is that non-missense mutations seem to be associated with an increased risk of any degree AVB, as compared to missense mutations, although this is not statistically significant (61% versus 35%; *p* = 0.055). In particular, the frequency of low-degree AVB is twice as high in patients with a non-missense mutation as in those with a missense mutation (61% versus 31%, respectively; *p* = 0.027). Non-missense mutation carriers develop cardiac conduction disorders earlier than missense mutation carriers [25,42,43]. A possible pathophysiological mechanism underlying this finding could be the haploinsufficiency of the cardiac Lamin A/C, which may promote the early apoptosis of the conduction system myocyte [44].

Non-significant differences were observed in the onset of musculoskeletal and cardiac manifestations (43 vs. 38 years, respectively, *p* value = 0.849). Musculoskeletal involvement was much less common in our sample than in previous investigations [26]: it was identified only in nine (17%) of our patients, and in three (5%) of whom, it was recognized as the first manifestation of the pathology, before any cardiological sign (Table 1).

The results of our work, which need to be confirmed by further studies on a larger population, agree with some previous studies and suggest avoiding the risk of underestimating missense variants. Furthermore, our findings underline the importance of evaluating the arrhythmic risk of each patient individually and integrating it using a multiparametric approach, in agreement with the 2023 ESC guidelines for the management of cardiomyopathies [13].

## 5. Study Limitations

In the current study, it was not possible to establish causality due to its observational design. Furthermore, data were collected on a rare disease from only one centre, leading to a small number of participants and, consequently, a possible type 2 statistical error.

The development of wide multicentre registries that contain clinical data on patient variants and prognosis can help in stratifying the individual risk of SCD and preventing an underestimation of disease severity.

Musculoskeletal involvement was much less common in our cohort, making our results difficult to compare with those from other studies.

## 6. Conclusions

Our study examined gene–phenotype correlations in patients with LMNA-related CMP, which is characterized by a high interindividual variability. In particular, we investigated the association between adverse outcomes and mutation type (missense versus non-missense). No statistically significant differences in cardiovascular adverse events or dilated cardiomyopathy prevalence were identified based on the type of mutation. This suggests that carriers of missense mutations should not be underestimated as belonging to the low-risk group.

Personalized arrhythmic risk stratification is crucial in identifying high-risk subjects for primary prevention ICD implantation in LMNA-related cardiomyopathy patients. Several studies have questioned whether exclusively non-missense mutations may lead to a poor prognosis.

Further investigations involving a larger population, with a careful analysis of gene– phenotype correlations among patients with LMNA-linked cardiomyopathy, could significantly enhance our understanding in this context. This would allow for the inclusion of some of the most unfavourable missense mutations and the variant location (upstream of the NLS) for arrhythmic risk stratification of patients to be considered in guidelines.

## Figures and Tables

**Table 1:** Descriptive analysis of baseline phenotypic and genetic features of 54 patients with *LMNA* mutations.

Characteristics	Value
Age, median [IQR]:	
Years at first clinical manifestation	41 [19]
Years at first clinical evaluation	45 [17] *p* value = 0.797
Age of disease onset (years), median [IQR]:	
Cardiac disease	38 [22]
Skeletal muscle disease	43 [33] *p* value = 0.849
Sex male, *n* (%)	29 (54)
Probands, *n* (%)	20 (37)
Relatives, *n* (%)	34 (63)
Years of follow-up, median [IQR]	8 [8]
Alive at last follow-up, *n* (%)	51 (94)
Family history for, *n* (%):	
Cardiomyopathy	45 (83)
SCD	29 (54)
Arrhythmias	30 (55)
Type of genetic mutation, *n* (%):	
Missense	26 (48)
Non-frameshift insertion	16 (30)
Frameshift deletion	5 (9)
Nonsense	7 (13)
Splicing site	0 (0)
Skeletal muscle disease	
At any time, *n* (%)	9 (17)
As first clinical manifestation, *n* (%)	3 (5)
Onset during the follow-up, *n* (%)	6 (11)
Specific phenotype, *n* (%):	
LGMD1B	2 (22)
EDMD2	0 (0)
LCMD	0 (0)
Non-specific phenotype, *n* (%)	7 (78)
Cardiac disease	
At any time, *n* (%)	44 (81)
As first clinical manifestation, *n* (%)	41 (76)
Onset during the follow-up, *n* (%)	3 (5)
Arrhythmic phenotype:	
AVB, *n* (%):	26 (48)
1st degree	25 (46)
2nd or 3rd degree	13 (24)
SVA (AF/AFL or tachycardia), *n* (%)	22 (41)
PVCs, *n* (%)	41 (76)
NSVT, *n* (%)	36 (67)
SVT, *n* (%)	16 (29)
VF, *n* (%)	1 (2)
appropriate ICD interventions, *n* (%)	17 (31)
Structural phenotype:	
LV dilation, *n* (%)	28 (52)
LV dysfunction, *n* (%):	
Mild	7 (13)
Moderate	8 (15)
Severe	12 (22)
RV involvement, *n* (%)	13 (24)
Combined arrhythmic and structural phenotype, *n* (%)	32 (59)
ICD carriers, *n* (%)	24 (44)
CRT-D carriers, *n* (%)	6 (11)

AF = atrial fibrillation; AFL = atrial flutter; AVB = atrioventricular block; CRT-D = cardiac resynchronization therapy with defibrillator; EDMD2 = type 2 Emery–Dreifuss muscular dystrophy; ICD = implantable cardioverter defibrillator; IQR = interquartile range; LCMD = LMNA-related congenital muscular dystrophy; LGMD1B = type 1B limb–girdle muscular dystrophy; LMNA = Lamin A/C gene; LV = left ventricular; NSVT = non-sustained ventricular tachycardia; PVCs = premature ventricular complexes; RV = right ventricular; SCD = sudden cardiac death; SVA = supraventricular arrhythmia; SVT = sustained ventricular tachycardia; and VF = ventricular fibrillation.

**Table 2:** Descriptive analysis of baseline phenotypic characteristics of patients by gender.

Type of Event	Male Sex (*n* = 30)	Female Sex (*n* = 24)	*p* Value	Odds Ratio (CI 95%) *
MACE, *n* (%)	13 (43.3)	5 (20.8)	0.081	2.91 (0.85–9.85)
Cardiac death, *n* (%)	2 (6.7)	1 (4.2)	0.690	1.64 (0.14–19.29)
Heart transplantation/ transplant waiting list, *n* (%)	6 (20.0)	2 (8.3)	0.230	2.75 (0.50–15.08)
Appropriate ICD intervention, *n* (%)	12 (40.0)	5 (20.8)	0.132	2.53 (0.74–8.64)
SVT, *n* (%)	11 (36.7)	5 (20.8)	0.205	2.20 (0.64–7.55)
NSVT, *n* (%)	22 (73.3)	13 (54.2)	0.143	2.33 (0.74–7.27)
PVCs, *n* (%)	23 (76.7)	18 (75.0)	0.887	1.09 (0.31–3.83)
SVA, *n* (%)	13 (43.3)	9 (37.5)	0.665	1.27 (0.42–3.82)
AF, *n* (%)	13 (43.3)	7 (29.2)	0.284	1.86 (0.59–5.80)
AFL, *n* (%)	2 (6.7)	3 (12.5)	0.462	0.50 (0.07–3.26)
AVB, *n* (%)	16 (53.3)	10 (41.7)	0.394	1.60 (0.54–4.73)
First-degree AVB, *n* (%)	15 (50)	10 (41.7)	0.542	1.40 (0.47–4.13)
Second- or third-degree AVB, *n* (%)	11 (36.7)	2 (8.3)	0.016	6.37 (1.25–32.40)
Dilated phenotype, *n* (%)	21 (70)	7 (29.2)	0.003	5.67 (1.75–18.38)
Mild LV dysfunction, *n* (%)	5 (16.7)	2 (8.7)	0.396	2.10 (0.37–11.96)
Moderate LV dysfunction, *n* (%)	5 (16.7)	3 (13.0)	0.715	1.33 (0.28–6.27)
Severe LV dysfunction, *n* (%)	9 (30.0)	3 (13.0)	0.144	2.86 (0.67–12.10)
RV involvement, *n* (%)	10 (34.5)	3 (13.6)	0.091	3.33 (0.79–14.05)

* Male sex was considered as reference. AF = atrial fibrillation; AFL = atrial flutter; AVB = atrioventricular block; ICD = implantable cardioverter defibrillator; LV = left ventricular; MACE = major adverse cardiovascular events; NSVT = non-sustained ventricular tachycardia; PVCs = premature ventricular complexes; RV = right ventricular; SVA = supraventricular arrhythmia; SVT = sustained ventricular tachycardia.

**Table 3:** Median age and IQR of the first occurrence of cardiovascular manifestations in patients with *LMNA* mutations.

Cardiovascular Manifestation	Median Age [IQR]
Cardiac death	57 [52–63]
Heart transplantation	55 [49–61]
Appropriate ICD interventions	53 [47–59]
1st degree AVB	41 [35–49]
2nd degree AVB	37 [35–49]
3rd degree AVB	54 [52–59]
AF	49 [43–53]
AFL	48 [43–50]
NSVT	48 [36–52]
SVT/VF	54 [47–59]
Dilated phenotype	50 [44–59]
Mild LV disfunction	48 [38–52]
Moderate LV disfunction	52 [47–58]
Severe LV disfunction	54 [47–60]
RV involvement	53 [44–59]

AF = atrial fibrillation; AFL = atrial flutter; AVB = atrioventricular block; ICD = implantable cardioverter defibrillator; IQR = interquartile range; *LMNA* = lamin A/C gene; LV = left ventricular; NSVT = non-sustained ventricular tachycardia; RV = right ventricular; SVT = sustained ventricular tachycardia; and VF = ventricular fibrillation.

**Table 4:** Comparison of cardiac manifestations related to non-missense vs. missense LMNA mutations.

Type of Event	Missense Mutation (*n* = 26)	Non-Missense Mutation (*n* = 28)	*p* Value	Odds Ratio (CI 95%) *
MACE, *n* (%)	9 (35)	9 (32)	0.847	0.89 (0.29–2.78)
Cardiac death, *n* (%)	2 (8)	1 (4)	0.509	0.44 (0.38–5.21)
Heart transplantation/ transplant waiting list, *n* (%)	4 (15)	4 (14)	1.000	0.92 (0.16–5.02)
appropriate ICD intervention, *n* (%)	9 (35)	8 (32)	0.847	0.895 (0.28–2.77)
SVT, *n* (%)	8 (31)	8 (29)	0.860	0.90 (0.28–2.89)
NSVT, *n* (%)	15 (58)	20 (71)	0.291	1.83 (0.59–5.67)
PVCs, *n* (%)	17 (65)	24 (86)	0.081	3.18 (0.84–12.03)
SVA, *n* (%)	9 (35)	13 (46)	0.377	0.61 (0.20–1.83)
AF, *n* (%)	9 (35)	11 (39)	0.723	1.63 (0.55–4.09)
AFL, *n* (%)	1 (4)	4 (14)	0.353	4.17 (0.43–40.00)
AVB, *n* (%)	9 (35)	17 (61)	0.055	2.92 (0.96–8.84)
First-degree AVB, *n* (%)	8 (31)	17 (61)	0.027	3.48 (1.12–10.73)
Second- or third-degree AVB, *n* (%)	4 (15)	9 (32)	0.150	0.38 (0.10–1.45)
Dilated phenotype, *n* (%)	16 (61)	12 (43)	0.170	0.47 (0.15–1.39)
Mild LV dysfunction, *n* (%)	2 (8)	5 (18)	0.426	2.50 (0.43–14.22)
Moderate LV dysfunction, *n* (%)	4 (15)	4 (14)	1.000	0.87 (0.19–3.94)
Severe LV dysfunction, *n* (%)	5 (19)	7 (25)	0.378	0.56 (0.15–2.06)
RV involvement, *n* (%)	6 (23)	9 (32)	0.378	1.78 (0.49–6.43)

* Non-missense mutations were considered as reference. AF = atrial fibrillation; AFL = atrial flutter; AVB = atrioventricular block; ICD = implantable cardioverter defibrillator; LV = left ventricular; MACE = major adverse cardiac events; NSVT = non-sustained ventricular tachycardia; PVCs = premature ventricular complexes; RV = right ventricular; SVA = supraventricular arrhythmia; SVT = sustained ventricular tachycardia.

## Data Availability

The anonymized data from this study are not publicly available due to privacy but can be made available from the corresponding author (C.F.) on request to qualified researchers who have obtained appropriate institutional review board (IRB) approval.
